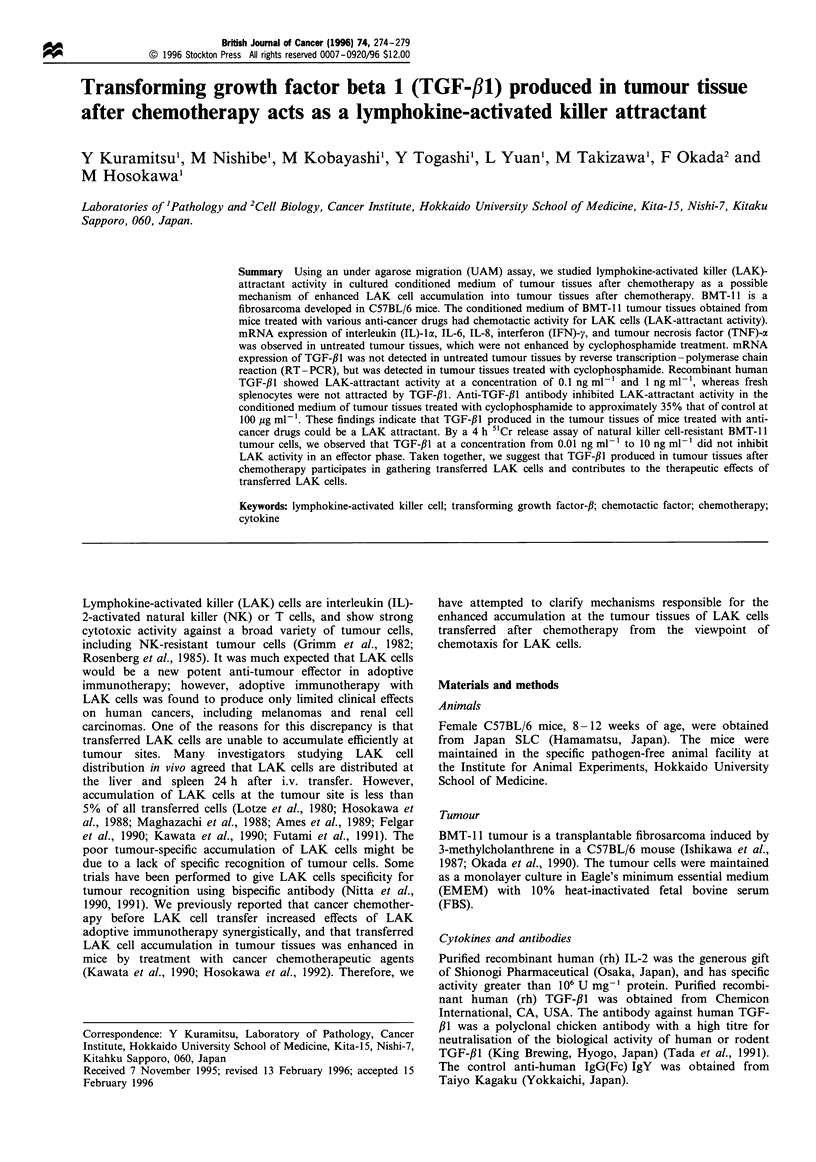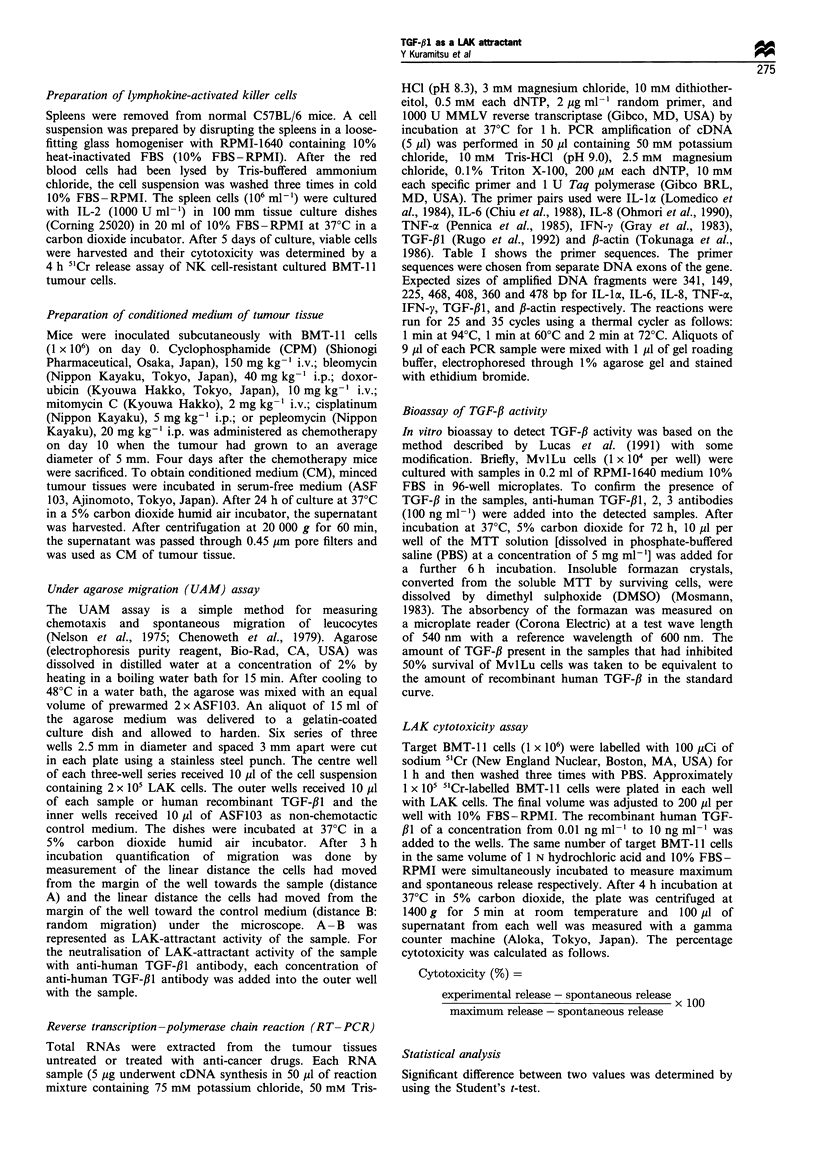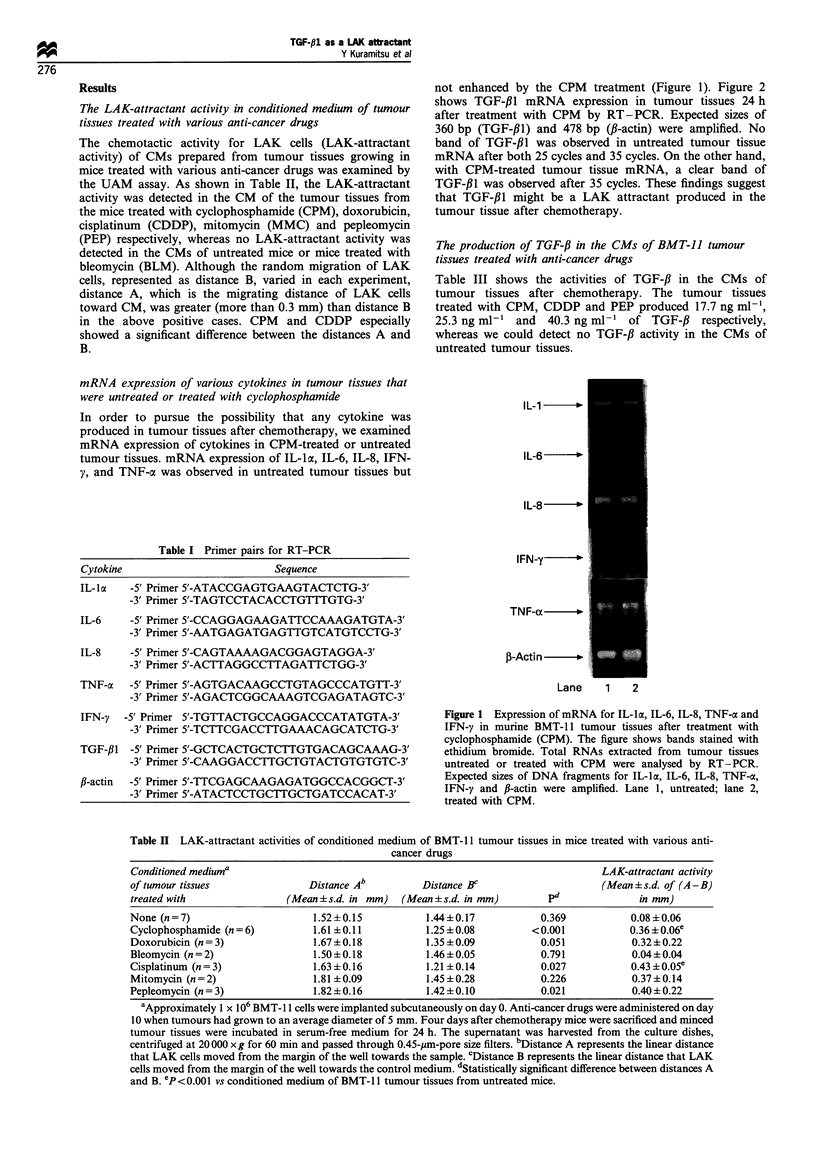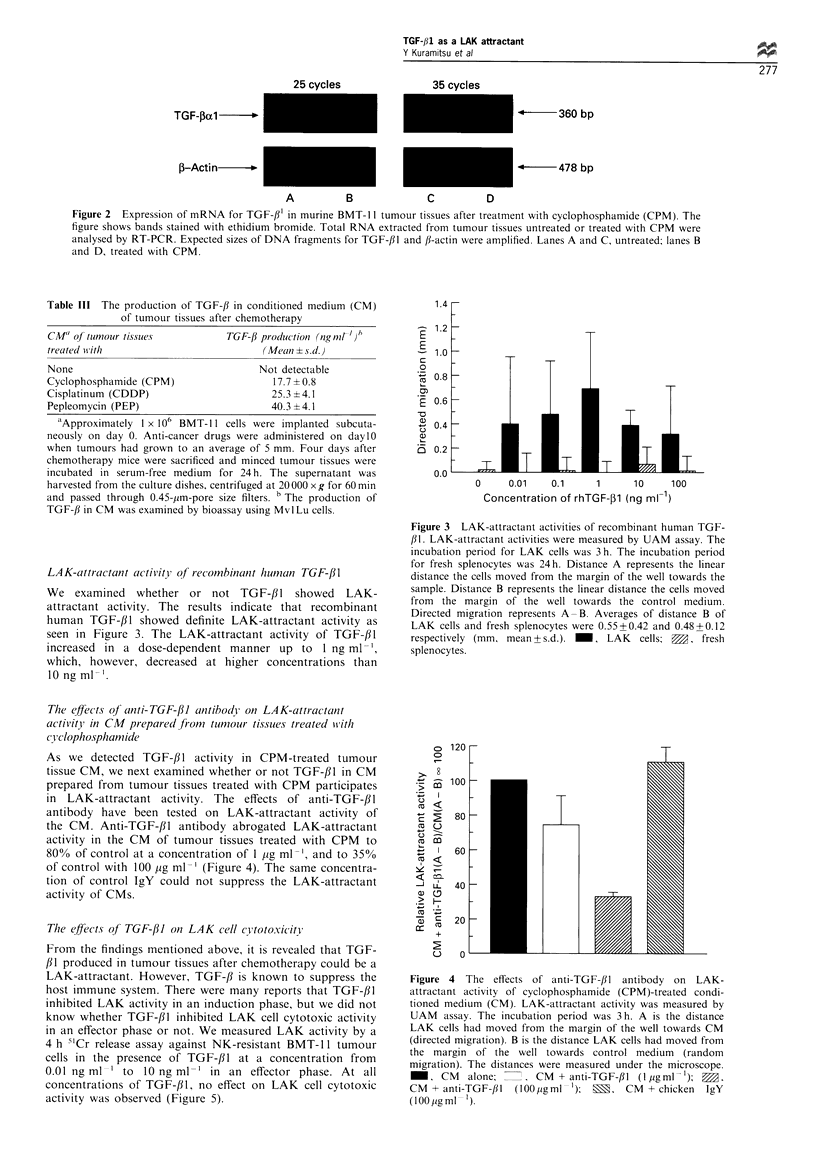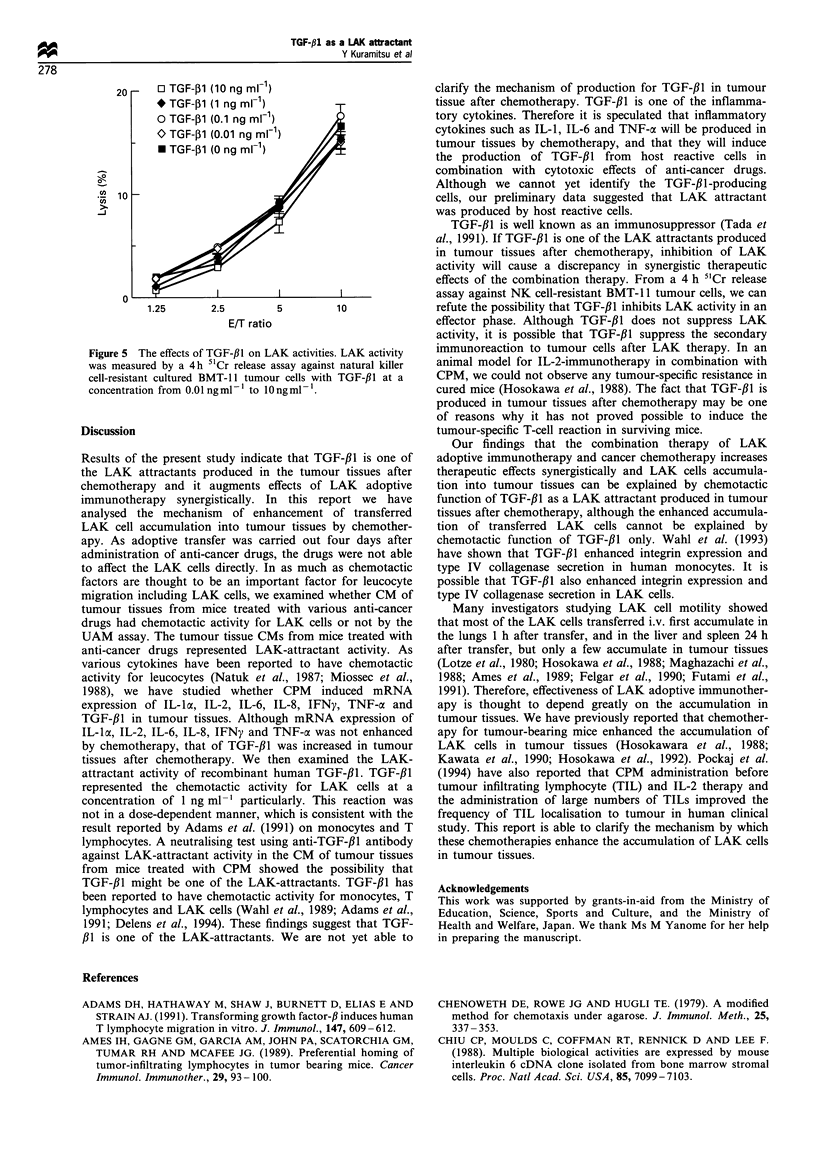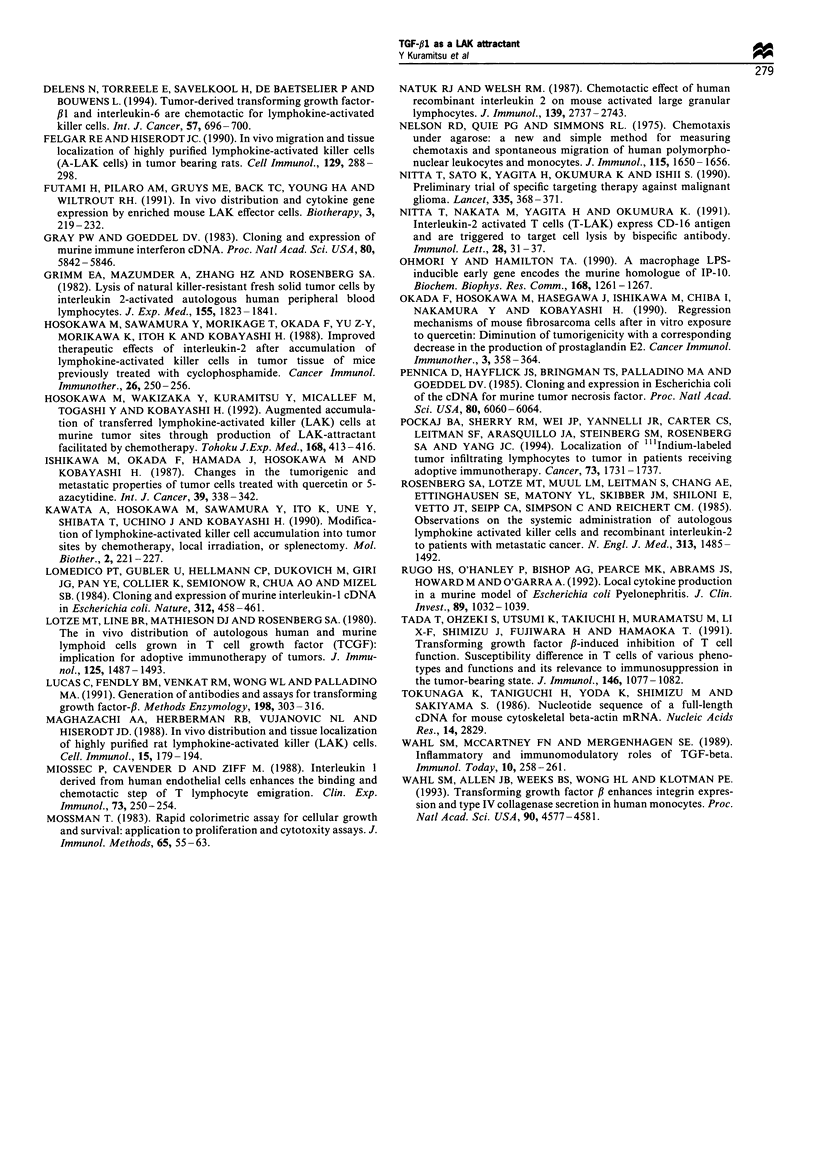# Transforming growth factor beta 1 (TGF-beta 1) produced in tumour tissue after chemotherapy acts as a lymphokine-activated killer attractant.

**DOI:** 10.1038/bjc.1996.351

**Published:** 1996-07

**Authors:** Y. Kuramitsu, M. Nishibe, M. Kobayashi, Y. Togashi, L. Yuan, M. Takizawa, F. Okada, M. Hosokawa

**Affiliations:** Laboratory of Pathology, Hokkaido University School of Medicine, Sapporo, Japan.

## Abstract

**Images:**


British Journal of Cancer (1996) 74, 274-279
© 1996 Stockton Press All rights reserved 0007-0920/96 $12.00

### Transforming growth factor beta 1 (TGF-β1) produced in tumour tissue after chemotherapy acts as a lymphokine-activated killer attractant

Y Kuramitsu[1], M Nishibe[1], M Kobayashi[1], Y Togashi[1], L Yuan[1], M Takizawa[1], F Okada[2] and M Hosokawa[1]

*Laboratories of [1]Pathology and [2]Cell Biology, Cancer Institute, Hokkaido University School of Medicine, Kita-15, Nishi-7, Kitaku Sapporo, 060, Japan.*

**Summary** Using an under agarose migration (UAM) assay, we studied lymphokine-activated killer (LAK)-attractant activity in cultured conditioned medium of tumour tissues after chemotherapy as a possible mechanism of enhanced LAK cell accumulation into tumour tissues after chemotherapy. BMT-11 is a fibrosarcoma developed in C57BL/6 mice. The conditioned medium of BMT-11 tumour tissues obtained from mice treated with various anti-cancer drugs had chemotactic activity for LAK cells (LAK-attractant activity). mRNA expression of interleukin (IL)-1α, IL-6, IL-8, interferon (IFN)-γ, and tumour necrosis factor (TNF)-α was observed in untreated tumour tissues, which were not enhanced by cyclophosphamide treatment. mRNA expression of TGF-β1 was not detected in untreated tumour tissues by reverse transcription-polymerase chain reaction (RT-PCR), but was detected in tumour tissues treated with cyclophosphamide. Recombinant human TGF-β1 showed LAK-attractant activity at a concentration of 0.1 ng $ml^{-1}$ and 1 ng $ml^{-1}$, whereas fresh splenocytes were not attracted by TGF-β1. Anti-TGF-β1 antibody inhibited LAK-attractant activity in the conditioned medium of tumour tissues treated with cyclophosphamide to approximately 35% that of control at 100 μg $ml^{-1}$. These findings indicate that TGF-β1 produced in the tumour tissues of mice treated with anti-cancer drugs could be a LAK attractant. By a 4 h $^{51}Cr$ release assay of natural killer cell-resistant BMT-11 tumour cells, we observed that TGF-β1 at a concentration from 0.01 ng $ml^{-1}$ to 10 ng $ml^{-1}$ did not inhibit LAK activity in an effector phase. Taken together, we suggest that TGF-β1 produced in tumour tissues after chemotherapy participates in gathering transferred LAK cells and contributes to the therapeutic effects of transferred LAK cells.

**Keywords:** lymphokine-activated killer cell; transforming growth factor-β; chemotactic factor; chemotherapy; cytokine

Lymphokine-activated killer (LAK) cells are interleukin (IL)-2-activated natural killer (NK) or T cells, and show strong cytotoxic activity against a broad variety of tumour cells, including NK-resistant tumour cells (Grimm *et al.*, 1982; Rosenberg *et al.*, 1985). It was much expected that LAK cells would be a new potent anti-tumour effector in adoptive immunotherapy; however, adoptive immunotherapy with LAK cells was found to produce only limited clinical effects on human cancers, including melanomas and renal cell carcinomas. One of the reasons for this discrepancy is that transferred LAK cells are unable to accumulate efficiently at tumour sites. Many investigators studying LAK cell distribution *in vivo* agreed that LAK cells are distributed at the liver and spleen 24 h after i.v. transfer. However, accumulation of LAK cells at the tumour site is less than 5% of all transferred cells (Lotze *et al.*, 1980; Hosokawa *et al.*, 1988; Maghazachi *et al.*, 1988; Ames *et al.*, 1989; Felgar *et al.*, 1990; Kawata *et al.*, 1990; Futami *et al.*, 1991). The poor tumour-specific accumulation of LAK cells might be due to a lack of specific recognition of tumour cells. Some trials have been performed to give LAK cells specificity for tumour recognition using bispecific antibody (Nitta *et al.*, 1990, 1991). We previously reported that cancer chemotherapy before LAK cell transfer increased effects of LAK adoptive immunotherapy synergistically, and that transferred LAK cell accumulation in tumour tissues was enhanced in mice by treatment with cancer chemotherapeutic agents (Kawata *et al.*, 1990; Hosokawa *et al.*, 1992). Therefore, we have attempted to clarify mechanisms responsible for the enhanced accumulation at the tumour tissues of LAK cells transferred after chemotherapy from the viewpoint of chemotaxis for LAK cells.

Correspondence: Y Kuramitsu, Laboratory of Pathology, Cancer Institute, Hokkaido University School of Medicine, Kita-15, Nishi-7, Kitahku Sapporo, 060, Japan
Received 7 November 1995; revised 13 February 1996; accepted 15 February 1996

### Materials and methods

### *Animals*

Female C57BL/6 mice, 8-12 weeks of age, were obtained from Japan SLC (Hamamatsu, Japan). The mice were maintained in the specific pathogen-free animal facility at the Institute for Animal Experiments, Hokkaido University School of Medicine.

### *Tumour*

BMT-11 tumour is a transplantable fibrosarcoma induced by 3-methylcholanthrene in a C57BL/6 mouse (Ishikawa *et al.*, 1987; Okada *et al.*, 1990). The tumour cells were maintained as a monolayer culture in Eagle's minimum essential medium (EMEM) with 10% heat-inactivated fetal bovine serum (FBS).

### *Cytokines and antibodies*

Purified recombinant human (rh) IL-2 was the generous gift of Shionogi Pharmaceutical (Osaka, Japan), and has specific activity greater than $10^6$ U $mg^{-1}$ protein. Purified recombinant human (rh) TGF-β1 was obtained from Chemicon International, CA, USA. The antibody against human TGF-β1 was a polyclonal chicken antibody with a high titre for neutralisation of the biological activity of human or rodent TGF-β1 (King Brewing, Hyogo, Japan) (Tada *et al.*, 1991). The control anti-human IgG(Fc) IgY was obtained from Taiyo Kagaku (Yokkaichi, Japan).

*Preparation of lymphokine-activated killer cells*

Spleens were removed from normal C57BL/6 mice. A cell suspension was prepared by disrupting the spleens in a loose-fitting glass homogeniser with RPMI-1640 containing 10% heat-inactivated FBS (10% FBS–RPMI). After the red blood cells had been lysed by Tris-buffered ammonium chloride, the cell suspension was washed three times in cold 10% FBS–RPMI. The spleen cells ($10^6$ $ml^{-1}$) were cultured with IL-2 (1000 U $ml^{-1}$) in 100 mm tissue culture dishes (Corning 25020) in 20 ml of 10% FBS–RPMI at 37°C in a carbon dioxide incubator. After 5 days of culture, viable cells were harvested and their cytotoxicity was determined by a 4 h $^{51}Cr$ release assay of NK cell-resistant cultured BMT-11 tumour cells.

*Preparation of conditioned medium of tumour tissue*

Mice were inoculated subcutaneously with BMT-11 cells ($1 \times 10^6$) on day 0. Cyclophosphamide (CPM) (Shionogi Pharmaceutical, Osaka, Japan), 150 mg $kg^{-1}$ i.v.; bleomycin (Nippon Kayaku, Tokyo, Japan), 40 mg $kg^{-1}$ i.p.; doxorubicin (Kyouwa Hakko, Tokyo, Japan), 10 mg $kg^{-1}$ i.v.; mitomycin C (Kyouwa Hakko), 2 mg $kg^{-1}$ i.v.; cisplatinum (Nippon Kayaku), 5 mg $kg^{-1}$ i.p.; or pepleomycin (Nippon Kayaku), 20 mg $kg^{-1}$ i.p. was administered as chemotherapy on day 10 when the tumour had grown to an average diameter of 5 mm. Four days after the chemotherapy mice were sacrificed. To obtain conditioned medium (CM), minced tumour tissues were incubated in serum-free medium (ASF 103, Ajinomoto, Tokyo, Japan). After 24 h of culture at 37°C in a 5% carbon dioxide humid air incubator, the supernatant was harvested. After centrifugation at 20 000 *g* for 60 min, the supernatant was passed through 0.45 $\mu$m pore filters and was used as CM of tumour tissue.

*Under agarose migration (UAM) assay*

The UAM assay is a simple method for measuring chemotaxis and spontaneous migration of leucocytes (Nelson *et al.*, 1975; Chenoweth *et al.*, 1979). Agarose (electrophoresis purity reagent, Bio-Rad, CA, USA) was dissolved in distilled water at a concentration of 2% by heating in a boiling water bath for 15 min. After cooling to 48°C in a water bath, the agarose was mixed with an equal volume of prewarmed 2 × ASF103. An aliquot of 15 ml of the agarose medium was delivered to a gelatin-coated culture dish and allowed to harden. Six series of three wells 2.5 mm in diameter and spaced 3 mm apart were cut in each plate using a stainless steel punch. The centre well of each three-well series received 10 $\mu$l of the cell suspension containing $2 \times 10^5$ LAK cells. The outer wells received 10 $\mu$l of each sample or human recombinant TGF-$\beta$1 and the inner wells received 10 $\mu$l of ASF103 as non-chemotactic control medium. The dishes were incubated at 37°C in a 5% carbon dioxide humid air incubator. After 3 h incubation quantification of migration was done by measurement of the linear distance the cells had moved from the margin of the well towards the sample (distance A) and the linear distance the cells had moved from the margin of the well toward the control medium (distance B: random migration) under the microscope. A–B was represented as LAK-attractant activity of the sample. For the neutralisation of LAK-attractant activity of the sample with anti-human TGF-$\beta$1 antibody, each concentration of anti-human TGF-$\beta$1 antibody was added into the outer well with the sample.

*Reverse transcription–polymerase chain reaction (RT–PCR)*

Total RNAs were extracted from the tumour tissues untreated or treated with anti-cancer drugs. Each RNA sample (5 $\mu$g underwent cDNA synthesis in 50 $\mu$l of reaction mixture containing 75 mM potassium chloride, 50 mM Tris-HCl (pH 8.3), 3 mM magnesium chloride, 10 mM dithiothereitol, 0.5 mM each dNTP, 2 $\mu$g $ml^{-1}$ random primer, and 1000 U MMLV reverse transcriptase (Gibco, MD, USA) by incubation at 37°C for 1 h. PCR amplification of cDNA (5 $\mu$l) was performed in 50 $\mu$l containing 50 mM potassium chloride, 10 mM Tris-HCl (pH 9.0), 2.5 mM magnesium chloride, 0.1% Triton X-100, 200 $\mu$M each dNTP, 10 mM each specific primer and 1 U *Taq* polymerase (Gibco BRL, MD, USA). The primer pairs used were IL-1$\alpha$ (Lomedico *et al.*, 1984), IL-6 (Chiu *et al.*, 1988), IL-8 (Ohmori *et al.*, 1990), TNF-$\alpha$ (Pennica *et al.*, 1985), IFN-$\gamma$ (Gray *et al.*, 1983), TGF-$\beta$1 (Rugo *et al.*, 1992) and $\beta$-actin (Tokunaga *et al.*, 1986). Table I shows the primer sequences. The primer sequences were chosen from separate DNA exons of the gene. Expected sizes of amplified DNA fragments were 341, 149, 225, 468, 408, 360 and 478 bp for IL-1$\alpha$, IL-6, IL-8, TNF-$\alpha$, IFN-$\gamma$, TGF-$\beta$1, and $\beta$-actin respectively. The reactions were run for 25 and 35 cycles using a thermal cycler as follows: 1 min at 94°C, 1 min at 60°C and 2 min at 72°C. Aliquots of 9 $\mu$l of each PCR sample were mixed with 1 $\mu$l of gel roading buffer, electrophoresed through 1% agarose gel and stained with ethidium bromide.

*Bioassay of TGF-$\beta$ activity*

*In vitro* bioassay to detect TGF-$\beta$ activity was based on the method described by Lucas *et al.* (1991) with some modification. Briefly, Mv1Lu cells ($1 \times 10^4$ per well) were cultured with samples in 0.2 ml of RPMI-1640 medium 10% FBS in 96-well microplates. To confirm the presence of TGF-$\beta$ in the samples, anti-human TGF-$\beta$1, 2, 3 antibodies (100 ng $ml^{-1}$) were added into the detected samples. After incubation at 37°C, 5% carbon dioxide for 72 h, 10 $\mu$l per well of the MTT solution [dissolved in phosphate-buffered saline (PBS) at a concentration of 5 mg $ml^{-1}$] was added for a further 6 h incubation. Insoluble formazan crystals, converted from the soluble MTT by surviving cells, were dissolved by dimethyl sulphoxide (DMSO) (Mosmann, 1983). The absorbency of the formazan was measured on a microplate reader (Corona Electric) at a test wave length of 540 nm with a reference wavelength of 600 nm. The amount of TGF-$\beta$ present in the samples that had inhibited 50% survival of Mv1Lu cells was taken to be equivalent to the amount of recombinant human TGF-$\beta$ in the standard curve.

*LAK cytotoxicity assay*

Target BMT-11 cells ($1 \times 10^6$) were labelled with 100 $\mu$Ci of sodium $^{51}Cr$ (New England Nuclear, Boston, MA, USA) for 1 h and then washed three times with PBS. Approximately $1 \times 10^5$ $^{51}Cr$-labelled BMT-11 cells were plated in each well with LAK cells. The final volume was adjusted to 200 $\mu$l per well with 10% FBS–RPMI. The recombinant human TGF-$\beta$1 of a concentration from 0.01 ng $ml^{-1}$ to 10 ng $ml^{-1}$ was added to the wells. The same number of target BMT-11 cells in the same volume of 1 N hydrochloric acid and 10% FBS–RPMI were simultaneously incubated to measure maximum and spontaneous release respectively. After 4 h incubation at 37°C in 5% carbon dioxide, the plate was centrifuged at 1400 *g* for 5 min at room temperature and 100 $\mu$l of supernatant from each well was measured with a gamma counter machine (Aloka, Tokyo, Japan). The percentage cytotoxicity was calculated as follows.

$$\text{Cytotoxicity (\%)} = \frac{\text{experimental release} - \text{spontaneous release}}{\text{maximum release} - \text{spontaneous release}} \times 100$$

*Statistical analysis*

Significant difference between two values was determined by using the Student's *t*-test.

### Results

*The LAK-attractant activity in conditioned medium of tumour tissues treated with various anti-cancer drugs*

The chemotactic activity for LAK cells (LAK-attractant activity) of CMs prepared from tumour tissues growing in mice treated with various anti-cancer drugs was examined by the UAM assay. As shown in Table II, the LAK-attractant activity was detected in the CM of the tumour tissues from the mice treated with cyclophosphamide (CPM), doxorubicin, cisplatinum (CDDP), mitomycin (MMC) and pepleomycin (PEP) respectively, whereas no LAK-attractant activity was detected in the CMs of untreated mice or mice treated with bleomycin (BLM). Although the random migration of LAK cells, represented as distance B, varied in each experiment, distance A, which is the migrating distance of LAK cells toward CM, was greater (more than 0.3 mm) than distance B in the above positive cases. CPM and CDDP especially showed a significant difference between the distances A and B.

*mRNA expression of various cytokines in tumour tissues that were untreated or treated with cyclophosphamide*

In order to pursue the possibility that any cytokine was produced in tumour tissues after chemotherapy, we examined mRNA expression of cytokines in CPM-treated or untreated tumour tissues. mRNA expression of IL-1α, IL-6, IL-8, IFN-γ, and TNF-α was observed in untreated tumour tissues but not enhanced by the CPM treatment (Figure 1). Figure 2 shows TGF-β1 mRNA expression in tumour tissues 24 h after treatment with CPM by RT–PCR. Expected sizes of 360 bp (TGF-β1) and 478 bp (β-actin) were amplified. No band of TGF-β1 was observed in untreated tumour tissue mRNA after both 25 cycles and 35 cycles. On the other hand, with CPM-treated tumour tissue mRNA, a clear band of TGF-β1 was observed after 35 cycles. These findings suggest that TGF-β1 might be a LAK attractant produced in the tumour tissue after chemotherapy.

*The production of TGF-β in the CMs of BMT-11 tumour tissues treated with anti-cancer drugs*

Table III shows the activities of TGF-β in the CMs of tumour tissues after chemotherapy. The tumour tissues treated with CPM, CDDP and PEP produced 17.7 ng $ml^{-1}$, 25.3 ng $ml^{-1}$ and 40.3 ng $ml^{-1}$ of TGF-β respectively, whereas we could detect no TGF-β activity in the CMs of untreated tumour tissues.

**Table I** Primer pairs for RT–PCR

| *Cytokine* | | *Sequence* |
|---|---|---|
| IL-1α | -5′ Primer | 5′-ATACCGAGTGAAGTACTCTG-3′ |
| | -3′ Primer | 5′-TAGTCCTACACCTGTTTGTG-3′ |
| IL-6 | -5′ Primer | 5′-CCAGGAGAAGATTCCAAAGATGTA-3′ |
| | -3′ Primer | 5′-AATGAGATGAGTTGTCATGTCCTG-3′ |
| IL-8 | -5′ Primer | 5′-CAGTAAAAGACGGAGTAGGA-3′ |
| | -3′ Primer | 5′-ACTTAGGCCTTAGATTCTGG-3′ |
| TNF-α | -5′ Primer | 5′-AGTGACAAGCCTGTAGCCCATGTT-3′ |
| | -3′ Primer | 5′-AGACTCGGCAAAGTCGAGATAGTC-3′ |
| IFN-γ | -5′ Primer | 5′-TGTTACTGCCAGGACCCATATGTA-3′ |
| | -3′ Primer | 5′-TCTTCGACCTTGAAACAGCATCTG-3′ |
| TGF-β1 | -5′ Primer | 5′-GCTCACTGCTCTTGTGACAGCAAAG-3′ |
| | -3′ Primer | 5′-CAAGGACCTTGCTGTACTGTGTGTC-3′ |
| β-actin | -5′ Primer | 5′-TTCGAGCAAGAGATGGCCACGGCT-3′ |
| | -3′ Primer | 5′-ATACTCCTGCTTGCTGATCCACAT-3′ |

**Figure 1** Expression of mRNA for IL-1α, IL-6, IL-8, TNF-α and IFN-γ in murine BMT-11 tumour tissues after treatment with cyclophosphamide (CPM). The figure shows bands stained with ethidium bromide. Total RNAs extracted from tumour tissues untreated or treated with CPM were analysed by RT–PCR. Expected sizes of DNA fragments for IL-1α, IL-6, IL-8, TNF-α, IFN-γ and β-actin were amplified. Lane 1, untreated; lane 2, treated with CPM.

**Table II** LAK-attractant activities of conditioned medium of BMT-11 tumour tissues in mice treated with various anti-cancer drugs

| *Conditioned medium[a] of tumour tissues treated with* | *Distance A[b] (Mean ± s.d. in mm)* | *Distance B[c] (Mean ± s.d. in mm)* | P[d] | *LAK-attractant activity (Mean ± s.d. of (A−B) in mm)* |
|---|---|---|---|---|
| None (*n* = 7) | 1.52 ± 0.15 | 1.44 ± 0.17 | 0.369 | 0.08 ± 0.06 |
| Cyclophosphamide (*n* = 6) | 1.61 ± 0.11 | 1.25 ± 0.08 | <0.001 | 0.36 ± 0.06[e] |
| Doxorubicin (*n* = 3) | 1.67 ± 0.18 | 1.35 ± 0.09 | 0.051 | 0.32 ± 0.22 |
| Bleomycin (*n* = 2) | 1.50 ± 0.18 | 1.46 ± 0.05 | 0.791 | 0.04 ± 0.04 |
| Cisplatinum (*n* = 3) | 1.63 ± 0.16 | 1.21 ± 0.14 | 0.027 | 0.43 ± 0.05[e] |
| Mitomycin (*n* = 2) | 1.81 ± 0.09 | 1.45 ± 0.28 | 0.226 | 0.37 ± 0.14 |
| Pepleomycin (*n* = 3) | 1.82 ± 0.16 | 1.42 ± 0.10 | 0.021 | 0.40 ± 0.22 |

[a]Approximately $1 \times 10^6$ BMT-11 cells were implanted subcutaneously on day 0. Anti-cancer drugs were administered on day 10 when tumours had grown to an average diameter of 5 mm. Four days after chemotherapy mice were sacrificed and minced tumour tissues were incubated in serum-free medium for 24 h. The supernatant was harvested from the culture dishes, centrifuged at 20 000 × *g* for 60 min and passed through 0.45-μm-pore size filters. [b]Distance A represents the linear distance that LAK cells moved from the margin of the well towards the sample. [c]Distance B represents the linear distance that LAK cells moved from the margin of the well towards the control medium. [d]Statistically significant difference between distances A and B. [e]$P < 0.001$ *vs* conditioned medium of BMT-11 tumour tissues from untreated mice.

**Figure 2** Expression of mRNA for TGF-β[1] in murine BMT-11 tumour tissues after treatment with cyclophosphamide (CPM). The figure shows bands stained with ethidium bromide. Total RNA extracted from tumour tissues untreated or treated with CPM were analysed by RT-PCR. Expected sizes of DNA fragments for TGF-β1 and β-actin were amplified. Lanes A and C, untreated; lanes B and D, treated with CPM.

**Table III** The production of TGF-β in conditioned medium (CM) of tumour tissues after chemotherapy

| *CM[a] of tumour tissues treated with* | *TGF-β production (ng ml$^{-1}$)[b] (Mean ± s.d.)* |
|---|---|
| None | Not detectable |
| Cyclophosphamide (CPM) | 17.7 ± 0.8 |
| Cisplatinum (CDDP) | 25.3 ± 4.1 |
| Pepleomycin (PEP) | 40.3 ± 4.1 |

[a]Approximately $1 \times 10^6$ BMT-11 cells were implanted subcutaneously on day 0. Anti-cancer drugs were administered on day10 when tumours had grown to an average of 5 mm. Four days after chemotherapy mice were sacrificed and minced tumour tissues were incubated in serum-free medium for 24 h. The supernatant was harvested from the culture dishes, centrifuged at 20 000 × *g* for 60 min and passed through 0.45-μm-pore size filters. [b] The production of TGF-β in CM was examined by bioassay using Mv1Lu cells.

*LAK-attractant activity of recombinant human TGF-β1*

We examined whether or not TGF-β1 showed LAK-attractant activity. The results indicate that recombinant human TGF-β1 showed definite LAK-attractant activity as seen in Figure 3. The LAK-attractant activity of TGF-β1 increased in a dose-dependent manner up to 1 ng ml$^{-1}$, which, however, decreased at higher concentrations than 10 ng ml$^{-1}$.

*The effects of anti-TGF-β1 antibody on LAK-attractant activity in CM prepared from tumour tissues treated with cyclophosphamide*

As we detected TGF-β1 activity in CPM-treated tumour tissue CM, we next examined whether or not TGF-β1 in CM prepared from tumour tissues treated with CPM participates in LAK-attractant activity. The effects of anti-TGF-β1 antibody have been tested on LAK-attractant activity of the CM. Anti-TGF-β1 antibody abrogated LAK-attractant activity in the CM of tumour tissues treated with CPM to 80% of control at a concentration of 1 μg ml$^{-1}$, and to 35% of control with 100 μg ml$^{-1}$ (Figure 4). The same concentration of control IgY could not suppress the LAK-attractant activity of CMs.

*The effects of TGF-β1 on LAK cell cytotoxicity*

From the findings mentioned above, it is revealed that TGF-β1 produced in tumour tissues after chemotherapy could be a LAK-attractant. However, TGF-β is known to suppress the host immune system. There were many reports that TGF-β1 inhibited LAK activity in an induction phase, but we did not know whether TGF-β1 inhibited LAK cell cytotoxic activity in an effector phase or not. We measured LAK activity by a 4 h $^{51}$Cr release assay against NK-resistant BMT-11 tumour cells in the presence of TGF-β1 at a concentration from 0.01 ng ml$^{-1}$ to 10 ng ml$^{-1}$ in an effector phase. At all concentrations of TGF-β1, no effect on LAK cell cytotoxic activity was observed (Figure 5).

**Figure 3** LAK-attractant activities of recombinant human TGF-β1. LAK-attractant activities were measured by UAM assay. The incubation period for LAK cells was 3 h. The incubation period for fresh splenocytes was 24 h. Distance A represents the linear distance the cells moved from the margin of the well towards the sample. Distance B represents the linear distance the cells moved from the margin of the well towards the control medium. Directed migration represents A – B. Averages of distance B of LAK cells and fresh splenocytes were 0.55 ± 0.42 and 0.48 ± 0.12 respectively (mm, mean ± s.d.). ■, LAK cells; ▨, fresh splenocytes.

**Figure 4** The effects of anti-TGF-β1 antibody on LAK-attractant activity of cyclophosphamide (CPM)-treated conditioned medium (CM). LAK-attractant activity was measured by UAM assay. The incubation period was 3 h. A is the distance LAK cells had moved from the margin of the well towards CM (directed migration). B is the distance LAK cells had moved from the margin of the well towards control medium (random migration). The distances were measured under the microscope. ■, CM alone; □, CM + anti-TGF-β1 (1 μg ml$^{-1}$); ▨, CM + anti-TGF-β1 (100 μg ml$^{-1}$); ▧, CM + chicken IgY (100 μg ml$^{-1}$).

**Figure 5** The effects of TGF-β1 on LAK activities. LAK activity was measured by a 4 h $^{51}$Cr release assay against natural killer cell-resistant cultured BMT-11 tumour cells with TGF-β1 at a concentration from 0.01 ng ml$^{-1}$ to 10 ng ml$^{-1}$.

### Discussion

Results of the present study indicate that TGF-β1 is one of the LAK attractants produced in the tumour tissues after chemotherapy and it augments effects of LAK adoptive immunotherapy synergistically. In this report we have analysed the mechanism of enhancement of transferred LAK cell accumulation into tumour tissues by chemotherapy. As adoptive transfer was carried out four days after administration of anti-cancer drugs, the drugs were not able to affect the LAK cells directly. In as much as chemotactic factors are thought to be an important factor for leucocyte migration including LAK cells, we examined whether CM of tumour tissues from mice treated with various anti-cancer drugs had chemotactic activity for LAK cells or not by the UAM assay. The tumour tissue CMs from mice treated with anti-cancer drugs represented LAK-attractant activity. As various cytokines have been reported to have chemotactic activity for leucocytes (Natuk *et al.*, 1987; Miossec *et al.*, 1988), we have studied whether CPM induced mRNA expression of IL-1α, IL-2, IL-6, IL-8, IFNγ, TNF-α and TGF-β1 in tumour tissues. Although mRNA expression of IL-1α, IL-2, IL-6, IL-8, IFNγ and TNF-α was not enhanced by chemotherapy, that of TGF-β1 was increased in tumour tissues after chemotherapy. We then examined the LAK-attractant activity of recombinant human TGF-β1. TGF-β1 represented the chemotactic activity for LAK cells at a concentration of 1 ng ml$^{-1}$ particularly. This reaction was not in a dose-dependent manner, which is consistent with the result reported by Adams *et al.* (1991) on monocytes and T lymphocytes. A neutralising test using anti-TGF-β1 antibody against LAK-attractant activity in the CM of tumour tissues from mice treated with CPM showed the possibility that TGF-β1 might be one of the LAK-attractants. TGF-β1 has been reported to have chemotactic activity for monocytes, T lymphocytes and LAK cells (Wahl *et al.*, 1989; Adams *et al.*, 1991; Delens *et al.*, 1994). These findings suggest that TGF-β1 is one of the LAK-attractants. We are not yet able to clarify the mechanism of production for TGF-β1 in tumour tissue after chemotherapy. TGF-β1 is one of the inflammatory cytokines. Therefore it is speculated that inflammatory cytokines such as IL-1, IL-6 and TNF-α will be produced in tumour tissues by chemotherapy, and that they will induce the production of TGF-β1 from host reactive cells in combination with cytotoxic effects of anti-cancer drugs. Although we cannot yet identify the TGF-β1-producing cells, our preliminary data suggested that LAK attractant was produced by host reactive cells.

TGF-β1 is well known as an immunosuppressor (Tada *et al.*, 1991). If TGF-β1 is one of the LAK attractants produced in tumour tissues after chemotherapy, inhibition of LAK activity will cause a discrepancy in synergistic therapeutic effects of the combination therapy. From a 4 h $^{51}$Cr release assay against NK cell-resistant BMT-11 tumour cells, we can refute the possibility that TGF-β1 inhibits LAK activity in an effector phase. Although TGF-β1 does not suppress LAK activity, it is possible that TGF-β1 suppress the secondary immunoreaction to tumour cells after LAK therapy. In an animal model for IL-2-immunotherapy in combination with CPM, we could not observe any tumour-specific resistance in cured mice (Hosokawa *et al.*, 1988). The fact that TGF-β1 is produced in tumour tissues after chemotherapy may be one of reasons why it has not proved possible to induce the tumour-specific T-cell reaction in surviving mice.

Our findings that the combination therapy of LAK adoptive immunotherapy and cancer chemotherapy increases therapeutic effects synergistically and LAK cells accumulation into tumour tissues can be explained by chemotactic function of TGF-β1 as a LAK attractant produced in tumour tissues after chemotherapy, although the enhanced accumulation of transferred LAK cells cannot be explained by chemotactic function of TGF-β1 only. Wahl *et al.* (1993) have shown that TGF-β1 enhanced integrin expression and type IV collagenase secretion in human monocytes. It is possible that TGF-β1 also enhanced integrin expression and type IV collagenase secretion in LAK cells.

Many investigators studying LAK cell motility showed that most of the LAK cells transferred i.v. first accumulate in the lungs 1 h after transfer, and in the liver and spleen 24 h after transfer, but only a few accumulate in tumour tissues (Lotze *et al.*, 1980; Hosokawa *et al.*, 1988; Maghazachi *et al.*, 1988; Ames *et al.*, 1989; Felgar *et al.*, 1990; Futami *et al.*, 1991). Therefore, effectiveness of LAK adoptive immunotherapy is thought to depend greatly on the accumulation in tumour tissues. We have previously reported that chemotherapy for tumour-bearing mice enhanced the accumulation of LAK cells in tumour tissues (Hosokawara *et al.*, 1988; Kawata *et al.*, 1990; Hosokawa *et al.*, 1992). Pockaj *et al.* (1994) have also reported that CPM administration before tumour infiltrating lymphocyte (TIL) and IL-2 therapy and the administration of large numbers of TILs improved the frequency of TIL localisation to tumour in human clinical study. This report is able to clarify the mechanism by which these chemotherapies enhance the accumulation of LAK cells in tumour tissues.

**Acknowledgements**
This work was supported by grants-in-aid from the Ministry of Education, Science, Sports and Culture, and the Ministry of Health and Welfare, Japan. We thank Ms M Yanome for her help in preparing the manuscript.

### References

ADAMS DH, HATHAWAY M, SHAW J, BURNETT D, ELIAS E AND STRAIN AJ. (1991). Transforming growth factor-β induces human T lymphocyte migration in vitro. *J. Immunol.*, **147**, 609–612.

AMES IH, GAGNE GM, GARCIA AM, JOHN PA, SCATORCHIA GM, TUMAR RH AND MCAFEE JG. (1989). Preferential homing of tumor-infiltrating lymphocytes in tumor bearing mice. *Cancer Immunol. Immunother.*, **29**, 93–100.

CHENOWETH DE, ROWE JG AND HUGLI TE. (1979). A modified method for chemotaxis under agarose. *J. Immunol. Meth.*, **25**, 337–353.

CHIU CP, MOULDS C, COFFMAN RT, RENNICK D AND LEE F. (1988). Multiple biological activities are expressed by mouse interleukin 6 cDNA clone isolated from bone marrow stromal cells. *Proc. Natl Acad. Sci. USA*, **85**, 7099–7103.

DELENS N, TORREELE E, SAVELKOOL H, DE BAETSELIER P AND BOUWENS L. (1994). Tumor-derived transforming growth factor-$\beta$1 and interleukin-6 are chemotactic for lymphokine-activated killer cells. *Int. J. Cancer*, **57**, 696–700.

FELGAR RE AND HISERODT JC. (1990). In vivo migration and tissue localization of highly purified lymphokine-activated killer cells (A-LAK cells) in tumor bearing rats. *Cell Immunol.*, **129**, 288–298.

FUTAMI H, PILARO AM, GRUYS ME, BACK TC, YOUNG HA AND WILTROUT RH. (1991). In vivo distribution and cytokine gene expression by enriched mouse LAK effector cells. *Biotherapy*, **3**, 219–232.

GRAY PW AND GOEDDEL DV. (1983). Cloning and expression of murine immune interferon cDNA. *Proc. Natl Acad. Sci. USA*, **80**, 5842–5846.

GRIMM EA, MAZUMDER A, ZHANG HZ AND ROSENBERG SA. (1982). Lysis of natural killer-resistant fresh solid tumor cells by interleukin 2-activated autologous human peripheral blood lymphocytes. *J. Exp. Med.*, **155**, 1823–1841.

HOSOKAWA M, SAWAMURA Y, MORIKAGE T, OKADA F, YU Z-Y, MORIKAWA K, ITOH K AND KOBAYASHI H. (1988). Improved therapeutic effects of interleukin-2 after accumulation of lymphokine-activated killer cells in tumor tissue of mice previously treated with cyclophosphamide. *Cancer Immunol. Immunother.*, **26**, 250–256.

HOSOKAWA M, WAKIZAKA Y, KURAMITSU Y, MICALLEF M, TOGASHI Y AND KOBAYASHI H. (1992). Augmented accumulation of transferred lymphokine-activated killer (LAK) cells at murine tumor sites through production of LAK-attractant facilitated by chemotherapy. *Tohoku J.Exp. Med.*, **168**, 413–416.

ISHIKAWA M, OKADA F, HAMADA J, HOSOKAWA M AND KOBAYASHI H. (1987). Changes in the tumorigenic and metastatic properties of tumor cells treated with quercetin or 5-azacytidine. *Int. J. Cancer*, **39**, 338–342.

KAWATA A, HOSOKAWA M, SAWAMURA Y, ITO K, UNE Y, SHIBATA T, UCHINO J AND KOBAYASHI H. (1990). Modification of lymphokine-activated killer cell accumulation into tumor sites by chemotherapy, local irradiation, or splenectomy. *Mol. Biother.*, **2**, 221–227.

LOMEDICO PT, GUBLER U, HELLMANN CP, DUKOVICH M, GIRI JG, PAN YE, COLLIER K, SEMIONOW R, CHUA AO AND MIZEL SB. (1984). Cloning and expression of murine interleukin-1 cDNA in *Escherichia coli*. *Nature*, **312**, 458–461.

LOTZE MT, LINE BR, MATHIESON DJ AND ROSENBERG SA. (1980). The in vivo distribution of autologous human and murine lymphoid cells grown in T cell growth factor (TCGF): implication for adoptive immunotherapy of tumors. *J. Immunol.*, **125**, 1487–1493.

LUCAS C, FENDLY BM, VENKAT RM, WONG WL AND PALLADINO MA. (1991). Generation of antibodies and assays for transforming growth factor-$\beta$. *Methods Enzymology*, **198**, 303–316.

MAGHAZACHI AA, HERBERMAN RB, VUJANOVIC NL AND HISERODT JD. (1988). In vivo distribution and tissue localization of highly purified rat lymphokine-activated killer (LAK) cells. *Cell. Immunol.*, **15**, 179–194.

MIOSSEC P, CAVENDER D AND ZIFF M. (1988). Interleukin 1 derived from human endothelial cells enhances the binding and chemotactic step of T lymphocyte emigration. *Clin. Exp. Immunol.*, **73**, 250–254.

MOSSMAN T. (1983). Rapid colorimetric assay for cellular growth and survival: application to proliferation and cytotoxity assays. *J. Immunol. Methods*, **65**, 55–63.

NATUK RJ AND WELSH RM. (1987). Chemotactic effect of human recombinant interleukin 2 on mouse activated large granular lymphocytes. *J. Immunol.*, **139**, 2737–2743.

NELSON RD, QUIE PG AND SIMMONS RL. (1975). Chemotaxis under agarose: a new and simple method for measuring chemotaxis and spontaneous migration of human polymorphonuclear leukocytes and monocytes. *J. Immunol.*, **115**, 1650–1656.

NITTA T, SATO K, YAGITA H, OKUMURA K AND ISHII S. (1990). Preliminary trial of specific targeting therapy against malignant glioma. *Lancet*, **335**, 368–371.

NITTA T, NAKATA M, YAGITA H AND OKUMURA K. (1991). Interleukin-2 activated T cells (T-LAK) express CD-16 antigen and are triggered to target cell lysis by bispecific antibody. *Immunol. Lett.*, **28**, 31–37.

OHMORI Y AND HAMILTON TA. (1990). A macrophage LPS-inducible early gene encodes the murine homologue of IP-10. *Biochem. Biophys. Res. Comm.*, **168**, 1261–1267.

OKADA F, HOSOKAWA M, HASEGAWA J, ISHIKAWA M, CHIBA I, NAKAMURA Y AND KOBAYASHI H. (1990). Regression mechanisms of mouse fibrosarcoma cells after in vitro exposure to quercetin: Diminution of tumorigenicity with a corresponding decrease in the production of prostaglandin E2. *Cancer Immunol. Immunother.*, **3**, 358–364.

PENNICA D, HAYFLICK JS, BRINGMAN TS, PALLADINO MA AND GOEDDEL DV. (1985). Cloning and expression in Escherichia coli of the cDNA for murine tumor necrosis factor. *Proc. Natl Acad. Sci. USA*, **80**, 6060–6064.

POCKAJ BA, SHERRY RM, WEI JP, YANNELLI JR, CARTER CS, LEITMAN SF, ARASQUILLO JA, STEINBERG SM, ROSENBERG SA AND YANG JC. (1994). Localization of $^{111}$Indium-labeled tumor infiltrating lymphocytes to tumor in patients receiving adoptive immunotherapy. *Cancer*, **73**, 1731–1737.

ROSENBERG SA, LOTZE MT, MUUL LM, LEITMAN S, CHANG AE, ETTINGHAUSEN SE, MATONY YL, SKIBBER JM, SHILONI E, VETTO JT, SEIPP CA, SIMPSON C AND REICHERT CM. (1985). Observations on the systemic administration of autologous lymphokine activated killer cells and recombinant interleukin-2 to patients with metastatic cancer. *N. Engl. J. Med.*, **313**, 1485–1492.

RUGO HS, O'HANLEY P, BISHOP AG, PEARCE MK, ABRAMS JS, HOWARD M AND O'GARRA A. (1992). Local cytokine production in a murine model of *Escherichia coli* Pyelonephritis. *J. Clin. Invest.*, **89**, 1032–1039.

TADA T, OHZEKI S, UTSUMI K, TAKIUCHI H, MURAMATSU M, LI X-F, SHIMIZU J, FUJIWARA H AND HAMAOKA T. (1991). Transforming growth factor $\beta$-induced inhibition of T cell function. Susceptibility difference in T cells of various phenotypes and functions and its relevance to immunosuppression in the tumor-bearing state. *J. Immunol.*, **146**, 1077–1082.

TOKUNAGA K, TANIGUCHI H, YODA K, SHIMIZU M AND SAKIYAMA S. (1986). Nucleotide sequence of a full-length cDNA for mouse cytoskeletal beta-actin mRNA. *Nucleic Acids Res.*, **14**, 2829.

WAHL SM, MCCARTNEY FN AND MERGENHAGEN SE. (1989). Inflammatory and immunomodulatory roles of TGF-beta. *Immunol. Today*, **10**, 258–261.

WAHL SM, ALLEN JB, WEEKS BS, WONG HL AND KLOTMAN PE. (1993). Transforming growth factor $\beta$ enhances integrin expression and type IV collagenase secretion in human monocytes. *Proc. Natl Acad. Sci. USA*, **90**, 4577–4581.